# Functional Characterization of Anthocyanin Biosynthesis-Related *Dihydroflavonol 4-reductase* (*DFR*) Genes in Blueberries (*Vaccinium corymbosum*)

**DOI:** 10.3390/plants14101449

**Published:** 2025-05-13

**Authors:** Yongyan Zhang, Sijian Guo, Zening Zhang, Ruide Li, Shitao Du, Siyi Hao, Chunzhen Cheng

**Affiliations:** College of Horticulture, Shanxi Agricultural University, Taigu 030801, China

**Keywords:** anthocyanin biosynthesis, blueberry, dihydroflavonol 4-reductase, promoter activation, transcriptional regulation

## Abstract

*Dihydroflavonol 4-reductase* (*DFR*) genes contribute greatly to anthocyanin biosynthesis in plants. Up to now, however, research on the *DFR* gene family and the key anthocyanin-related *DFR* members in blueberries (*Vaccinium corymbosum*) has been limited. In this study, we performed a genome-wide identification of the blueberry *DFR* gene family, identifying 36 *VcDFR* genes categorized into five subfamilies. Gene expression analysis showed that three Subfamily III members (*VcDFR11/29/34*) and four Subfamily V members (*VcDFR4/7/30/33*) are highly expressed in blueberry fruits, particularly at late ripening stages. Transient overexpression analysis in apple fruits verified the contributions of *VcDFR11* and *VcDFR30* to anthocyanin biosynthesis, with *VcDFR11* showing better promoting effects. Blueberry fruit-based transient overexpression further confirmed the promoting effects of *VcDFR11* on anthocyanin accumulation and the expression of anthocyanin-related structural genes (especially its downstream *anthocyanindin synthase* (*ANS*) and *UDP-glucose: flavonoid 3-O-glycosyltransferase* (*UFGT*) genes). The *VcDFR11* promoter contains binding sites for both bHLH and MYB transcription factors (TFs). Consistently, yeast one-hybrid and dual-luciferase assays confirmed that anthocyanin-related VcMYB-1 and VcbHLHs can bind to and activate the *VcDFR11* promoter. Furthermore, co-overexpressing *VcMYB-1*/*VcbHLHs* with *VcDFR11* led to much higher anthocyanin accumulation than overexpressing *VcDFR11* alone, indicating that these TFs positively regulate anthocyanin biosynthesis by upregulating *VcDFR11*. In summary, our study characterized the blueberry *DFR* gene family and demonstrated the role of *VcDFR11* in anthocyanin biosynthesis.

## 1. Introduction

Dihydroflavonol 4-reductase (DFR, EC1.1.1.219) catalyzes the conversion of dihydroflavonols to leucoanthocyanidins and is the first committed enzyme of the anthocyanin/proanthocyanidin biosynthesis pathway [1,2,3]. In recent years, *DFR* genes involved in anthocyanin biosynthesis have been identified and functionally characterized in numerous plant species [4,5,6]. For example, the expression level of pomegranate (*Punica granatum* L.) *PgDFR* is positively correlated with anthocyanin accumulation, and its expression in pomegranates with black skin is approximately 3- and 5-fold higher than in samples with red or green skin [4], respectively. In leaves of different *Malus crabapple* cultivars the expression of *McDFR1* is positively correlated with the anthocyanin content [5]. In pink-leaved ornamental kale (*Brassica oleracea* var. *acephala*) the expression of *BoDFR1* is very significantly higher than in the white-leaved line, and its silencing in the pink-leaved line leads to significantly reduced anthocyanin accumulation [6]. Evidence has revealed that mutations in *DFR* genes will cause abnormal anthocyanin accumulation in plants [6,7,8]. For instance, the Arabidopsis *DFR* mutant (*tt3*) plant shows no anthocyanidins accumulation in seeds and vegetative tissues [7]. In green-leaved kale, *BoDFR1* and *BoDFR2*, have a 1 bp and 2 bp insertion compared to pink-leaved kale [9], respectively. Recently, the functions of several plant *DFRs* have been verified through genetic transformation analysis [6,10,11]. The heterologous overexpression of *AtDFR* in *Brassica napus* L. significantly increases anthocyanin accumulation [10]. *Camellia sinensis CsDFRa* and *CsDFRc* overexpression can restore the abnormal anthocyanin accumulation phenotypes of *tt3* Arabidopsis plants [11]. Moreover, overexpression of *B. oleracea BoDFR1* in white-leaved kale results in obvious anthocyanin accumulation [6].

Many transcription factors (TFs) have been reported to play important regulatory roles in plant anthocyanin biosynthesis and accumulation by regulating *DFR* transcription [12,13]. *M. crabapple* McMYB10 positively regulates anthocyanin biosynthesis by binding to and activating the promoter of *McDFR1* and upregulating its expression [5]. In table grapes (*Vitis vinifera* L.), VvMYB23 and VvbHLH93 can bind to and activate the promoter of *VvDFR*, and upregulate its expression [14]. *Dendrobium officinale* DoMYB5 and DobHLH24 can both activate the promoter of *DoDFR* [15]. In apples, MdMYB1 has been reported to have the ability of binding to the *MdDFR* promoter and regulating anthocyanin biosynthesis in the pericarp [16], while MdMYB10 regulates anthocyanin biosynthesis in apple pulp by interacting with MdbHLH3/33 to improve the activity of the *MdDFR* promoter [17]. The interaction between MdERF38 and MdMYB1 greatly enhances the binding ability of MdMYB1 to the promoters of *MdDFR* and *MdUF3GT*, thereby increasing anthocyanin accumulation [18]. In peach (*Prunus persica*), PpMYB108 binds to the promoter of *PpDFR* and plays important roles in regulating anthocyanin accumulation in the flower [19]; and PpMYB75 functions in promoting flesh pigmentation by binding to the ‘CCGTTG’ sequence in the *PpDFR* promoter and upregulating its expression [20]. In addition to TFs acting as activators of anthocyanin biosynthesis, some TFs negatively regulate anthocyanin accumulation by downregulating *DFR* [21,22]. For example, potato (*Solanum tuberosum* L.) StMYB44 negatively regulates anthocyanin biosynthesis in tuber flesh via binding to and suppressing the promoter of *StDFR*, thereby downregulating its expression [21].

Blueberry (*Vaccinium corymbosum*) is a popular economic fruit crop celebrated for its anthocyanin-rich, highly nutritional and health-beneficial blue fruits [23,24,25]. There have been some studies on the role of DFR in blueberry anthocyanin biosynthesis [26]. For example, it was reported that both pre- and post-harvest UV irradiation can upregulate the expression of *VcDFR* and some other anthocyanin-related structural genes and increase anthocyanin accumulation in blueberry fruits [27]. The anthocyanin-related VcMYB1 can bind to and activate the promoter of *VcDFR* and positively regulate blueberry anthocyanin biosynthesis [28]. VcSnRK2.3 can interact with VcMYB1 and enhance the activation ability of VcMYB1 on the *VcDFR* promoter [29]. Research demonstrates that plant DFRs are encoded by a gene family and that different DFR members have various functions [30]. However, up to now, there has been no report focusing on the systematic analysis of the *DFR* gene family in blueberries. In 2015, Gupta et al. [31] published the draft genome of a blueberry, which can greatly facilitate the systematic identification and characterization of anthocyanin-related genes. In this study, we performed genome-wide identification and characterization analyses of the *VcDFR* gene family, and functionally characterized two *VcDFRs* (*VcDFR11* and *VcDFR30*) that were highly expressed in blueberry fruits. Furthermore, the binding abilities of anthocyanin-related TFs (VcMYB-1 [32] and four VcbHLHs [33]) to the *VcDFR11* promoter were studied by using yeast one-hybrid (Y1H) and dual-luciferase (LUC) assays. Additionally, to verify the possible ‘TF-DFR’ modules involving in anthocyanin biosynthesis, apple fruit-based transient overexpression was performed by co-expressing *VcDFR11* with anthocyanin-related *TFs*. Our study will be helpful for understanding the characteristics of the blueberry *DFRs* and will provide a basis for clarifying the roles of DFR in anthocyanin biosynthesis.

## 2. Results

### 2.1. Identification and Physiochemical Properties Analyses Results of VcDFRs

**Figure 1:**
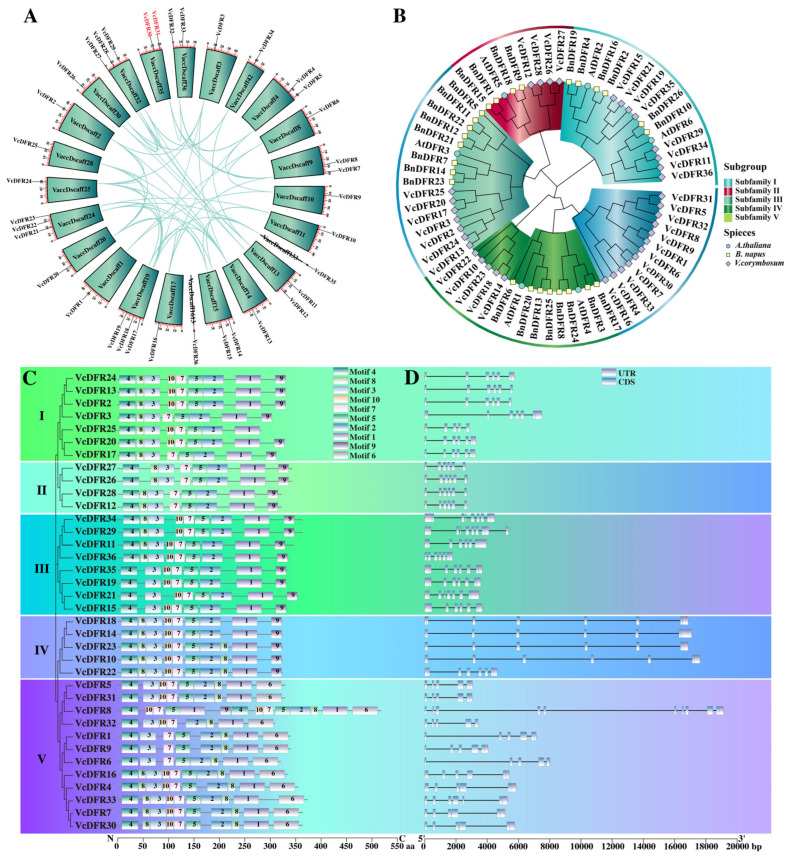
Synteny (**A**), phylogenetic (**B**), conserved motif (**C**), and gene structure (**D**) analysis results of VcDFRs. (**A**) Distribution and collinearity analysis results of *VcDFRs*. The lines represent duplicated *VcDFR* gene pairs, and the *VcDFRs* in red are tandemly duplicated gene pairs. (**B**) Phylogenetic tree constructed using DFRs from blueberries and some other plant species. Vc: *Vaccinium corymbosum*; At: *Arabidopsis thaliana*; Bn: *Brassica napus*. (**C**) Conserved motif analysis results of VcDFRs; N: the N-terminal; C: the C-terminal. aa: amino acid. (**D**) Gene structure analysis results of *VcDFRs*. UTR: untranslated region; CDS: coding sequence; bp: base pair.

In total, we identified 36 *VcDFRs* from the blueberry genome data. According to their chromosome (scaffold) localization information, they were named as *VcDFR1*~*VcDFR36* (Figure 1A). Their encoded proteins were of 282~518 aa, with molecular weight ranging from 31.44~56.33 kDa, and pI ranging from 5.36~8.59 (Supplementary Table S1). Subcellular localization prediction results revealed that 26 (72.22%), 6 (16.67%), 2 (5.56%), 1 (2.78%), and 1 (2.78%) VcDFRs were localized in Golgi apparatus, chloroplast and Golgi apparatus, cytoplasm, chloroplast and cytoplasm, and chloroplast, respectively.

### 2.2. Phylogenetic and Sequence Similarity Analyses Results of VcDFRs

Phylogenetic analysis of the DFRs from the blueberries *Arabidopsis thaliana* and *B. napus* successfully categorized them into five subfamilies (Figure 1B). Subfamily I contains seven VcDFRs, eight BnDFRs, and AtDFR3. Subfamily II consists of four VcDFRs, four BnDFRs, and AtDFR5. Subfamily III contains eight VcDFRs, six BnDFRs, and two AtDFRs (AtDFR2 and AtDFR6), among which VcDFR11, VcDFR29, and VcDFR34 are most closely related to AtDFR6. Moreover, all three VcDFRs share more than 70% similarity with AtDFR6 (74.53%, 70.59%, and 70.59%, respectively). Subfamily IV consists of five VcDFRs, eight BnDFRs, and two AtDFRs (AtDFR1 and AtDFR4). However, Subfamily V contains only twelve VcDFRs, indicating that this subfamily is blueberry specific. Sequence alignment results showed that the nucleotide similarities among the 36 *VcDFRs* ranged from 34.86% to 100% (Figure S1A), with high similarities ranging from 91.76% to 100% and from 87.31% to 100% among Subfamily II and IV members, respectively. The protein similarities of VcDFR members belonging to the five subfamilies ranged from 72.26% to 100%, 92.71% to 100%, 55.31% to 100%, 90.43% to 100%, and 45.84% to 99.72% (Figure S1B), respectively.

### 2.3. Synteny, Conserved Motif, and Gene Structure Analyses Results of VcDFRs

Synteny analysis identified 51 duplicated gene pairs involving 34 *VcDFRs* (except the Subfamily III member *VcDFR36* and the Subfamily IV member *VcDFR22*) (Figure 1A and Supplementary Table S3), including 1 tandem duplication gene pair (*VcDFR30* and *VcDFR31*) and 50 segmental duplication gene pairs. The Ka/Ks values of duplicated gene pairs range from 0 to 0.7146. The divergence of these duplicated gene pairs was calculated to occur at 0 million years ago (Mya) to 3.30 Mya.

In total, we identified 10 kinds of conserved motifs in VcDFRs (Figure 1C). Of them, motif1, motif2, motif4, and motif7 are contained by all VcDFRs. There are 35 (except VcDFR8), 35 (except DFR32), 34 (except VcDFR25 and VcDFR8), and 32 VcDFRs (except VcDFR15, VcDFR19, VcDFR21, and VcDFR35) containing motif3, motif5, motif9, and motif8, respectively. All Subfamily III and IV members contain motif10. Interestingly, motif6 is found to be Subfamily V-specific.

Gene structure analysis results showed that the intron numbers of *VcDFRs* ranged from 4~9 (Figure 1D). Except for one Subfamily I member (*VcDFR25*, containing four introns) and three Subfamily III members (*VcDFR21*, *VcDFR29*, and *VcDFR34*, containing six introns), all other Subfamily I~IV members contain five introns. Most of the Subfamily V members, however, contain four introns.

### 2.4. Promoter Analysis Results

**Figure 2:**
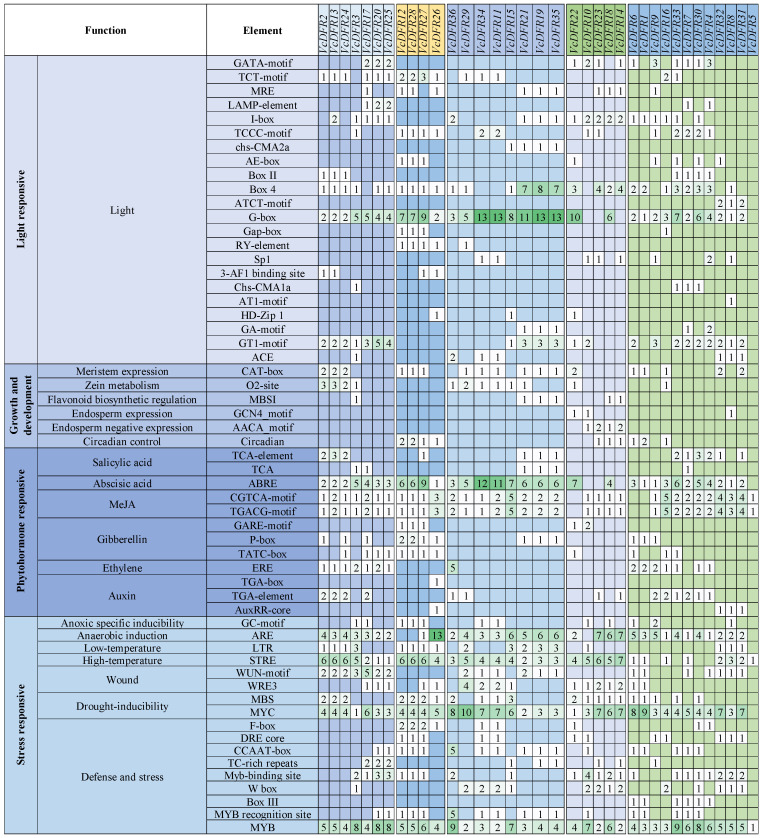
Predicted *cis*-acting elements in promoters of *VcDFRs*. The numbers in the boxes represent the number of different kinds of *cis*-elements identified in each *VcDFR* promoter.

*Cis*-acting elements prediction results revealed that *VcDFRs* promoters contained many light-, phytohormone-, stress-responsive-, and growth and development-related elements (Figure 2). In total, we identified 17 kinds of light-responsive elements in *VcDFRs* promoters. Of the light-responsive elements, G-box element (which is also a ubiquitous regulatory DNA element that can be bound by numerous TFs, such as bHLH and bZIP) was identified in 31 (86.11%) *VcDFRs* promoters. There were 33 (91.67%), 31 (86.11%), 18 (50%), 18 (50%), 15 (41.67%), and 15 (41.67%) *VcDFRs* containing methyl jasmonate (MeJA)-, abscisic acid (ABA)-, gibberellin (GA)-, auxin-, ethylene (ET)-, and salicylic acid (SA)-responsive elements in their promoters. Notably, all of the Subfamily I members contain the ET-responsive element ERE and all of the Subfamily I, II and III members contain MeJA- and ABA-responsive elements in their promoters. There are 32 (88.89%), 32 (88.89%), 28 (77.78%), 17 (47.22%), and 12 (33.33%) *VcDFRs* which contain anaerobic-induction-, high-temperature-, wound-, low-temperature-, and anoxic-specific-induction-related elements in their promoters, respectively. Additionally, meristem expression-, zein metabolism-, flavonoid biosynthetic regulation- and endosperm negative expression-related elements were found in the promoters of some *VcDFRs*.

**Figure 3:**
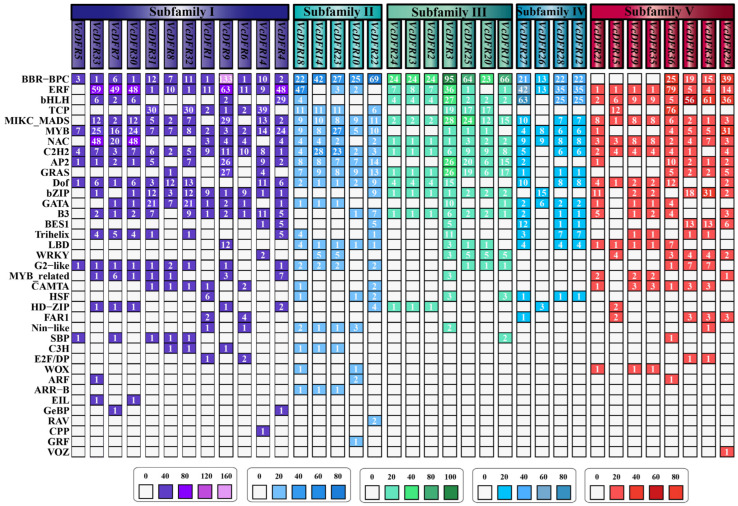
Predicted transcription factor binding sites (TFBSs) in promoters of *VcDFRs*. The numbers in the boxes represent the amount of predicted binding sites for different kinds of transcription factors. The number of TFBSs identified in promoters of *VcDFR* members from the five subfamilies are displayed in different colors.

The transcription factor binding sites (TFBSs) in *VcDFRs* promoters were further predicted (Figure 3). In total, the binding sites for 37 types of TFs are identified in *VcDFRs* promoters. Among them, C2H2 binding sites are identified in the largest number of *VcDFRs* promoters (34), followed by BCR-BPC (32), ERF (32), and MIKC_MADS (32). It is worth noting that all Subfamily II, IV, and V members have MYB binding sites, all Subfamily I and III members have bHLH binding sites, all Subfamily II and III members contain ERF binding sites, and all Subfamily I~III members have NAC binding sites in their promoters.

### 2.5. Gene Expression Analysis of VcDFRs

**Figure 4:**
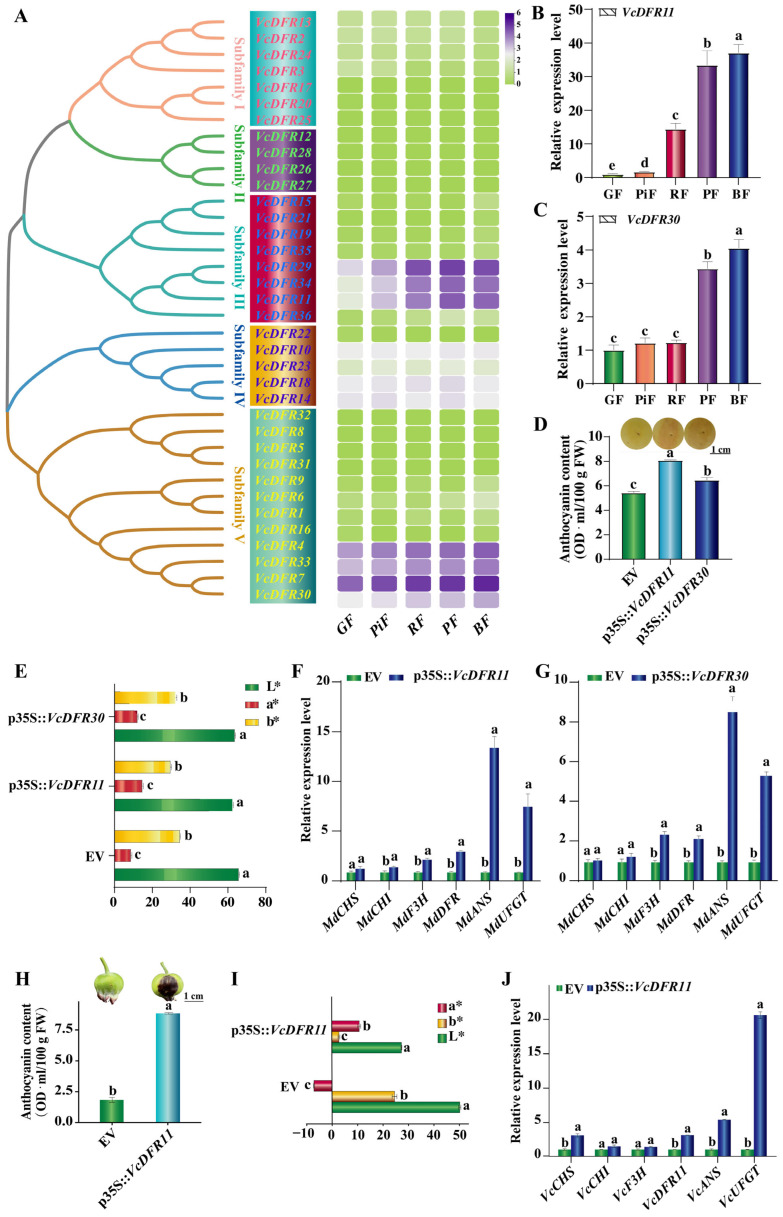
Gene expression and functional characterization analysis results of *VcDFRs*. (**A**): Transcriptome data-based gene expression analysis of *VcDFRs*. The heatmap is drawn using Log_2_(FPKM+1) values of *VcDFRs*. The redder the color, the higher the gene’s expression, and the greener the color, the lower the gene’s expression. (**B**,**C**): qRT-PCR analysis results for *VcDFR11* and *VcDFR30*, respectively. GF: green fruit; PiF: pink fruit; RF: red fruit; PF: purple fruit; BF: blue fruit. (**D**): Anthocyanin contents in apple peels. FW: fresh weight. Scale bar = 1 cm. (**E**): Color parameters of apple peels overexpressing EV, *VcDFR11*, and *VcDFR30*. (**F**,**G**): Effects of *VcDFR11* and *VcDFR30* transient overexpression on the expression of anthocyanin-biosynthesis structural genes in apple peels, respectively. Different lowercase letters above columns represent significant difference at *p* < 0.05 level. (**H**): Anthocyanin contents in blueberry peels. Scale bar = 1 cm. (**I**): Color parameters of blueberry peels overexpressing EV and *VcDFR11*. (**J**): Effects of *VcDFR11* transient overexpression on the expression of anthocyanin-biosynthesis structural genes in blueberry peels. Different letters above columns represent significant difference at *p* < 0.05 level. The L*, a*, and b* in (**E**,**I**) indicates value for lightness [from 0 (black) to 100 (white)]; red/green (+a* = redder, −a* = greener); and yellow/blue (+b* = yellower, −b* = bluer), respectively.

The expression patterns of *VcDFRs* in blueberry fruits at five ripening stages were first analyzed according to our transcriptome data (Figure 4A). Only three Subfamily III (*VcDFR11/29/34*, sharing nucleotide similarity > 94%), four Subfamily IV (*VcDFR18/14/10/23*, similarity > 85%), and four Subfamily V (*VcDFR4/7/30/33*, similarity > 86%, the similarity between *VcDFR7* and *VcDFR30* is 98.90%) members express in blueberry fruits at all stages. Of them, Subfamily III and V *VcDFR* members express much higher than Subfamily IV members, and their expression levels in red fruit (RF), purple fruit (PF), and blue fruit (BF) are much higher than that in green fruit (GF) and pink fruit (PiF) (Figure 4A).

Given the high similarities among the fruit expressed *VcDFRs* from the same subfamily, we further investigated the expression patterns of two fruit highly expressed VcDFRs, *VcDFR11* and *VcDFR30*, in blueberry fruits using quantitative real-time PCR (qRT-PCR) (Figure 4B,C). Consistent with the transcriptome data, our qRT-PCR results showed that the expression levels of these two *VcDFRs* in PF and BF were significantly higher than those in GF, PiF, and RF. The expression of *VcDFR11* in PiF, RF, PF, and BF was 1.73-, 14.47-, 33.46-, and 37.09-fold of GF, respectively. The expression of *VcDFR30* in PF and BF was 3.44- and 4.05-fold of GF, respectively.

### 2.6. Effects of Transient Overexpression of VcDFR11 and VcDFR30 on Anthocyanin Accumulations

By using apple fruit-based transient overexpression, we further studied the functions of *VcDFR11* and *VcDFR30*. Seven days post agrobacteria inoculation, apples overexpressing *VcDFR11* and *VcDFR30* showed obvious pigmentation in inoculated areas (Figure 4D). Compared to the apple fruit overexpressing the pBI121-GFP empty vector control (EV), the anthocyanin content in peels of apple fruit overexpressing *VcDFR11* and *VcDFR30* increased by 48.77% and 18.97%, respectively (Figure 4D). Notably, the anthocyanin content in apple peels overexpressing *VcDFR11* was found to be significantly higher than that overexpressing *VcDFR30*. By determining the color parameters of apple fruits, we found that the L* and b* values of apple peels overexpressing *VcDFR11* were the lowest, while their a* values were the highest (Figure 4E). *VcDFR11* overexpression significantly upregulated the expression of *MdCHI*, *MdF3H*, *MdDFR*, *MdANS*, and *MdUFGT* in apple peels (accounting for approximately 1.54-, 2.29-, 3.08-, 13.51-, and 7.60-fold of EV, respectively) (Figure 4F). Meanwhile, *VcDFR30* overexpression only upregulated the expression of *MdF3H*, *MdDFR*, *MdANS*, and *MdUFGT* in apple peels (accounting for approximately 2.40-, 2.94-, 12.57-, and 7.02-fold of EV, respectively) (Figure 4G). The promoting effects of *VcDFR11* on anthocyanin accumulation and the expression of most anthocyanin-biosynthesis-related structural genes are better than that of *VcDFR30*. These results indicated that the *VcDFR11* exhibited better fruit-pigmentation- and anthocyanin-biosynthesis-promoting effects than *VcDFR30*. It is worth noting that the two *VcDFRs*’ overexpression both led to a much larger multiple of upregulation of the downstream structural genes (*MdANS* and *MdUFGT*) than upstream genes of *DFR*, indicating that their overexpression greatly activated the downstream anthocyanin biosynthesis.

The function of *VcDFR11* was further studied by using blueberry fruit-based transient overexpression analysis. Results showed that the gene’s overexpression accelerated greatly the fruit peel pigmentation in inoculated areas, and the anthocyanin content in fruit peels overexpressing *VcDFR11* increased to about 4.88-fold of EV (Figure 4H). Similarly to the results obtained in apples, blueberry peels overexpressing *VcDFR11* had lower L* and b* values and a higher a* value than that overexpressing EV (Figure 4I). Moreover, *VcDFR11* overexpression upregulated the expression levels of *VcCHS*, *VcCHI*, *VcF3H*, *VcDFR11*, *VcANS*, and *VcUFGT* to 3.07-, 1.47-, 1.39-, 3.01-, 5.35-, and 20.68-fold of EV (Figure 4J), respectively. Consistent with the results obtained in apples, *VcDFR11* overexpression also led to a much larger multiple of upregulation of *VcANS* and *VcUFGT* genes in blueberry peels.

### 2.7. Anthocyanin-Related VcbHLHs and VcMYB-1 Can Bind to and Active the VcDFR11 Promoter

**Figure 5:**
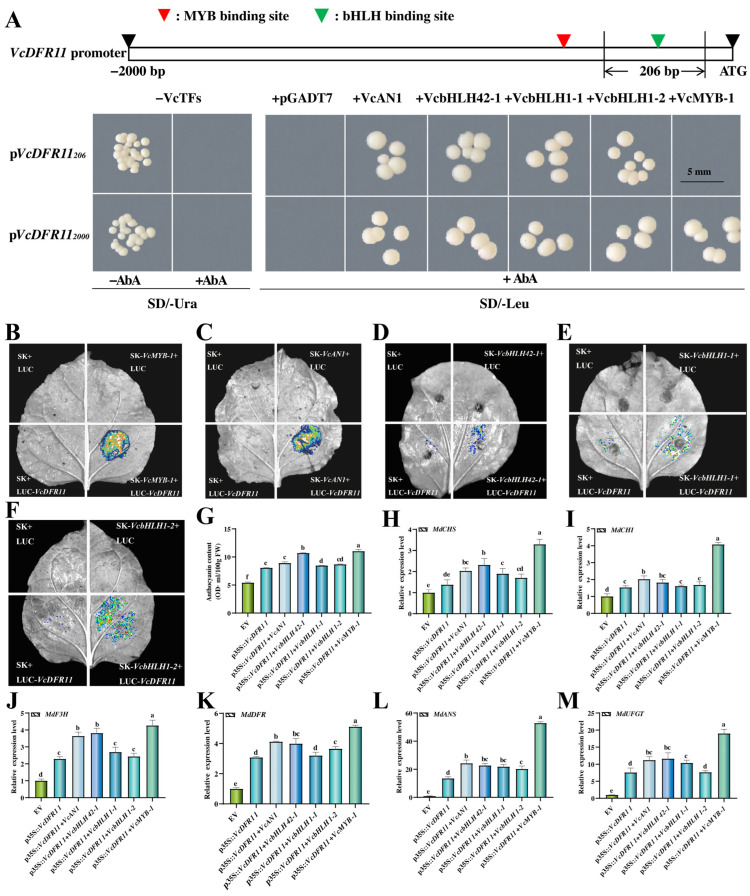
Regulations of anthocyanin-related transcription factors on *VcDFR11* and anthocyanin metabolism. (**A**): Diagram for the *VcDFR11* promoter sequence and Y1H validation results of the binding ability of VcAN1, VcbHLH42-1, VcbHLH1-1, VcbHLH1-2, and VcMYB-1 to the *VcDFR11* promoter. The VcDFR11_206_ promoter contains only bHLH binding sites, and the VcDFR11_2000_ promoter contains both bHLH and MYB binding sites. (**B**–**F**): LUC validation results for the binding activity of VcMYB-1, VcAN1, VcbHLH42-1, VcbHLH1-1, and VcbHLH1-2 to the *VcDFR11* promoter, respectively. SK and LUC represent the pNC-Green-SK and the pNC-Green-LUC empty vector, respectively. (**G**): Effects of transient overexpression of *VcbHLHs*, *VcMYB-1*, and *VcDFR11* on anthocyanin accumulation in apple peels. (**H**–**M**): Influences of the co-expression of *VcDFR11* and anthocyanin-related *TFs* (*VcAN1*, *VcbHLH42-1*, *VcbHLH1-1*, *VcbHLH1-2*, and *VcMYB-1*) on the expression of anthocyanin-biosynthesis-related structural genes (*MdCHS*, *MdCHI*, *MdF3H*, *MdDFR*, *MdANS*, and *MdUFGT*) in apple peels, respectively. Different lowercase letters above columns represent significant difference among samples at *p* < 0.05 level.

Promoter sequencing results showed that there were bHLH and MYB binding sites on the *VcDFR11* promoter, suggesting that its expression might be regulated by these two types of TFs. By using yeast one-hybrid assay (Y1H), the binding ability of anthocyanin-related VcAN1, VcbHLH42-1, VcbHLH1-1, VcbHLH1-2, and VcMYB-1 to the *VcDFR11* promoter was first studied. Results showed that the growth of Y1HGold yeast strains transformed with pVcDFR11_206_/pVcDFR11_2000_ was inhibited on SD/-Ura plates supplemented with AbA (Figure 5A), indicating that pVcDFR11_206_ and pVcDFR11_2000_ could not be self-activated. The yeast strains co-transformed with pVcDFR11_206_ and pGADT7/pGADT7-VcMYB-1, and pVcDFR11_2000_ and pGADT7 could not grow on SD/-Leu medium containing AbA. Meanwhile, yeast strains co-transformed with pVcDFR11_206_/pVcDFR11_2000_ and pGADT7-VcAN1/VcbHLH42-1/VcbHLH1-1/VcbHLH1-2/VcMYB-1 grew well. These results indicated that VcMYB-1 and the four VcbHLHs can bind to the *VcDFR* promoter. Consistently, our dual-luciferase (LUC) assay results also showed that VcMYB-1 and all the four anthocyanin-related VcbHLHs can enhance the fluorescence of *VcDFR11* promoter, indicating that they can bind to and activate the promoter of *VcDFR11* (Figure 5B–F). Notably, the binding and activation activities of VcMYB-1 and VcAN1 to the *VcDFR11* promoter were much stronger than other VcbHLHs. In one of our previous studies we have verified the interaction between VcMYB-1 and VcAN1 [25]. In this study, by using a firefly luciferase complementation imaging (LCI) assay the interactions between VcMYB-1 and VcbHLH42-1/VcbHLH1-1/VcbHLH1-2 were also verified (Figure S2), suggesting that the ‘MYB-bHLH-DFR’ module contributes greatly to the anthocyanin biosynthesis in blueberry.

### 2.8. Co-Overexpression of Anthocyanin-Related TFs with VcDFR11 Exhibited Better Anthocyanin Accumulaiton-Promoting Effects than Overexpressing VcDFR11 Alone

Transient overexpression experiments were further conducted to investigate the influences of anthocyanin-related *TFs* and *VcDFR11* co-overexpression on anthocyanin accumulation. Results showed that their co-expression resulted in significantly higher anthocyanin accumulations in apple peels than that overexpressing *VcDFR11* alone (Figure 5G). qRT-PCR analysis showed that the co-transformation of *VcDFR11* with *VcAN1*, *VcbHLH42-1*, *VcbHLH1-1*, *VcbHLH1-2*, and *VcMYB-1* further significantly upregulated the expression levels of anthocyanin-biosynthesis structural genes, particular *MdANS* and *MdUFGT* (with much higher fold changes than other structural genes), in apple peels (Figure 5H–M). Among them, the co-expression of *VcMYB-1* and *VcDFR11* exhibited the best promoting effect on anthocyanin accumulation and structural genes’ expression in apple fruit peels, followed by *VcAN1*/*VcbHLH42-1*.

## 3. Discussion

In this study, we identified 36 *DFR* genes from the blueberry genome, which was more than most other plants, such as Arabidopsis, *B. napus* [34], tea plant [11], and *Freesia hybrida* [35]. In *B. napus*, the expansion of the *DFR* gene family was caused by segmental and tandem duplications [34]. Similarly, we identified 50 segmental and 1 tandem duplicated *VcDFR* gene pairs in blueberry, indicating that segmental and tandem duplications, especially segmental duplications, promoted the expansion of the blueberry *DFR* gene family.

Of the 36 *VcDFRs*, three Subfamily III and four Subfamily V *VcDFRs* were expressed highly in fruits. It is worth noting that the three Subfamily III *VcDFRs* shared a close relationship with the anthocyanin-related *AtDFR6* [10,11]. Among them, *VcDFR11* encodes a protein sharing the highest similarity with AtDFR6 (74.53%). The transcriptional levels of *DFR* genes are highly correlated with the accumulation of anthocyanin in plants [36,37]. Our study found that almost all fruit highly expressed *VcDFRs* had higher expression at late ripening stages. Moreover, the *VcDFR11* and *VcDFR30* overexpression significantly increased anthocyanin accumulation and upregulated anthocyanin-biosynthesis-related structural genes, particularly *ANS* and *UFGT* genes. The *VcDFR11* reported in our study shared a 99.52% similarity with the *VcDFR* reported by Wang et al. [29], indicating that *VcDFR11* is a key *DFR* involved in anthocyanin biosynthesis in blueberry. Although the promoting effects of *VcDFR30* on anthocyanin biosynthesis are milder than that of *VcDFR11*, its overexpression also significantly improves anthocyanin accumulation and the corresponding structural genes’ expression. Therefore, it can be concluded that fruit highly expressed Subfamily III and V *VcDFRs* play important roles in the anthocyanin biosynthesis of blueberries.

The expression of anthocyanin-related genes is influenced by environmental factors and transcription factors [38,39]. Many studies have revealed that the high accumulation of flavonoids/anthocyanin under stress conditions, which results from activated *DFR* expression or its overexpression, is correlated with the stress tolerance of plants [10,40,41]. In this study, we identified many phytohormone- and stress-responsive elements in *VcDFRs* promoters, indicating that the expression of *VcDFRs* is influenced by a variety of phytohormones and environmental factors and that they might play important roles in blueberry stress responses.

Evidence has revealed that TFs, such as MYB and bHLH, participate in anthocyanin-biosynthesis regulation by binding to and activating/suppressing promoters of anthocyanin-biosynthesis-related structural genes [42,43], including *DFR*. Lu et al. [14] found that VvMYB3 and VvbHLH93 functioned in anthocyanin accumulation regulation in grape flesh by binding to the promoter of *VvDFR* and upregulating its transcription. Liu et al. [44] found that E3 ubiquitin ligase BoMIEL1 mediates the degradation of BoMYB4b, thereby alleviating the inhibitory effect of BoMYB4b on the *BoDFR1* promoter and promoting anthocyanin accumulation in kale. In this study, it was found that all Subfamily II, IV, and V *VcDFR* members process MYB binding sites on their promoters. Additionally, all Subfamily I and III *VcDFR* promoters have bHLH binding sites. Notably, both bHLH and MYB binding sites are present in the *VcDFR11* promoter. Our Y1H and LUC analysis results showed that anthocyanin-related VcbHLHs and VcMYB-1, especially VcAN1 and VcMYB-1, could bind to and activate the *VcDFR11* promoter. The co-overexpression of their encoding genes with *VcDFR11* led to a higher accumulation of anthocyanins than overexpressing *VcDFR11* did alone. Specifically, the co-overexpression of *VcMYB-1* and *VcDFR11* showed the best promoting effect on anthocyanin accumulation, followed by *VcAN1* and *VcbHLH42-1*. Future studies should further investigate their regulation on *VcDFR11*.

## 4. Materials and Methods

### 4.1. Plant Materials

Green, pink, red, purple, and blue fruits used in this study were harvested from two-year-old ‘FL03’ plants. For apple and blueberry fruit-based transient overexpression analysis, bagged ‘Gala’ apples and green ‘Legacy’ blueberry fruits were used. For LUC and LCI assays, six-week-old *Nicotiana benthamiana* plants were used.

### 4.2. Identification of Blueberry DFR Proteins

Arabidopsis DFR sequences (downloaded from TAIR, https://www.arabidopsis.org (accessed on 26 February 2024)) were used as queries to BLASTp against blueberry genome data (downloaded from GDV, https://www.vaccinium.org/analysis/49/ (accessed on 1 March 2024)) (E-value ≤ l × 10^−5^, identity ≥ 50%) [45]. Then, CDD (https://www.ncbi.nlm.nih.gov/Structure/cdd/wrpsb.cgi (accessed on 1 March 2024)) was used to verify the presence of the DFR domain (pfam01370) in candidate DFRs. Sequences containing complete DFR domain remained, with an exception of VaccDscaff12-snap-gene-66.28 whose coding sequence (CDS) was found to be quite different from the reference sequence in the genome data by gene cloning and sequencing validations (its CDS was only 1011 bp and had a very high similarity (>98.22%) with the VaccDscaff1613-processed gene-0.0 sequence).

### 4.3. Physiochemical Property, Subcellular Localization, and Phylogenetic Analyses of VcDFRs

The ExPASy—ProtParam tool (https://web.expasy.org/protparam/ (accessed on 1 March 2024)) and Plant-mPLoc (http://www.csbio.sjtu.edu.cn/bioinf/plant-multi/ (accessed on 1 March 2024)) [46] were used to predict the physiochemical properties and subcellular localization of VcDFRs, respectively. MEGA11 was first used to align the DFRs from blueberries, *Brassica napus*, and Arabidopsis. Then, a phylogenetic tree was constructed using the neighbor-joining method (bootstrap = 1000 and other parameters set as default). For the visualization of the phylogenetic tree, the ggtree package in R was used.

### 4.4. Synteny, Gene Structure and Conserved Motif Analyses

Based on the genome sequence and annotation data of blueberries, MCScanX was used to analyze the syntenies among the 36 *VcDFRs*, and ‘Circos’ of TBtools software v2.056 was used for figure drawing [47]. The simple Ka/Ks calculator (NG) program in TBtools software v2.056 was used to calculate the divergence time (T) of duplicated *VcDFR* gene pairs [48]. GSDS (http://gsds.cbi.pku.edu.cn/ (accessed on 1 March 2024)) and MEME (https://meme-suite.org/meme/index.html (accessed on 1 March 2024)) were used to analyze the gene structures and conserved motifs in their encoded proteins, respectively. TBtools was used for the figure drawing of gene structures and conserved motifs analyses results [49].

### 4.5. Promoter Analysis

The 2000 bp sequences upstream of the start codon (ATG) of *VcDFRs* were extracted from blueberry genome data using TBtools, and used as promoter sequences. For the prediction analysis of *cis*-acting elements and transcription factor binding sites in *VcDFRs* promoters, PlantCARE (http://bioinformatics.psb.ugent.be/webtools/plantcare/html/ (accessed on 1 March 2024)) and PlantTFDB (http://planttfdb.cbi.pku.edu.cn/ (accessed on 1 March 2024)) were used (*p*-value ≤ l × 10^−5^), respectively. Subsequently, an R package was utilized to show the distribution of *cis*-acting elements and transcription factor binding sites across the *VcDFRs* promoters.

### 4.6. RNA Isolation and Gene Cloning

Using an RNAprep pure plant kit (Cat#GDP432) (Tiangen, Beijing, China), we isolated total RNA samples of ‘FL03’ blueberry fruits at five ripening stages. The RevertAid first-strand cDNA synthesis kit (Catalog# K1622) (Thermo Scientific, Shanghai, China) was used for the biosynthesis of cDNA used for gene cloning, with equally weighted mixed fruit RNA as the template. Then, *VcDFR11* and *VcDFR30* CDSs were amplified using reverse transcription PCR (RT-PCR). The 20 μL RT-PCR system contains 10.0 μL Dream Taq™ green PCR master mix (2×), 7.0 μL ddH_2_O, 1 μL cDNA, and 1 μL each of forward and reverse primers. The amplification conditions were initial denaturation at 95 °C for 3 min, followed by 35 cycles (95 °C for 30 s, 56 °C for 30 s, 72 °C for 1 min), and final extension at 72 °C for 10 min. Target PCR products were gel purified and sent to Qingke Biotechnology (Fuzhou, China) Co., Ltd. for sequencing verification.

### 4.7. Gene Expression Analysis

To study the expression patterns of *VcDFRs* during fruit ripening, transcriptome data of ‘FL03’ blueberry fruits at green, pink, red, pink, and blue stages were first used. Then, quantitative real-time PCR (qRT-PCR) was used to verify the expression patterns of two highly expressed *VcDFRs*, *VcDFR11* and *VcDFR30*, in blueberry fruits at the five ripening stages. An RNAprep pure kit (Tiangen, Beijing, China) was used to isolate total RNA from fruits. High-quality blueberry fruit RNA was reverse transcribed into complementary DNA (cDNA) using the PrimeScript RT reagent kit with gDNA eraser (perfect real-time) kit (Cat# RR047A) (Takara, Dalian, China). For the designing of the primers used for the qRT-PCR analysis of *VcDFR11* and *VcDFR30*, Primer3web version 4.1.0 (https://primer3.ut.ee/) was used. qRT-PCR reactions were conducted on a QuantStudio 3 real-time PCR system (Applied Biosystems, Shanghai, China) with *GAPDH* (AY123769) as the internal reference gene [25]. The relative expression levels of *VcDFR11* and *VcDFR30* in different fruit samples were calculated using the 2^−ΔΔCt^ method [50]. For the gene expression analysis, three biological replications were used.

### 4.8. Vector Construction and Transient Overexpression Assays in Apple and Blueberry Fruits

According to Zhang et al. [32], the full-length CDSs of *VcDFR11* and *VcDFR30* (1038 bp and 1092 bp, respectively) were introduced into the pBI121-GFP vector to obtain p35S::VcDFR11 and p35S::VcDFR30 recombinant vectors, respectively. Primers used for vector construction are all listed in Supplementary Table S2. The *VcMYB-1* and *VcbHLHs* overexpression vectors used for co-expression experiments were provided by our lab [33,51].

After transforming overexpression vectors into *Agrobacterium tumefaciens* GV3101 component cells, infiltration solutions were prepared and injected into ‘Gala’ apple fruits [51,52] and green ‘Legacy’ blueberry fruits. For the transient overexpression of each vector, at least eight apple/blueberry fruits were used. After injection, fruits were kept in dark for 2 d and then removed to normal light condition at 25 °C for 5 d. The influences of the transient overexpression of selected *VcDFR* genes or gene combinations on fruit coloration changes were observed and photographed. A CR8 colorimeter (3nh, Guangzhou, China) was used to measure the color parameters of apple and blueberry fruits.

The anthocyanin contents in apple and blueberry fruit peels around the injection areas were determined. Briefly, fruit peels were homogenized with acidified ethanol, placed in the dark at approximately 25 °C for 1 d, and centrifuged at 10,000 rpm for 15 min to collect the supernatant. Then, the absorbance value of the supernatant solution was measured at 535 nm using a spectrophotometer (UV-1800, Shanghai Meixi Instrument Co., Ltd., Shanghai, China) and used for the calculation of the anthocyanin contents according to Yang et al. [53]. To investigate the influences of the *VcDFRs* overexpression on the expression of anthocyanin-biosynthesis-related structural genes, the relative expression levels of *MdCHS*, *MdCHI*, *MdF3H*, *MdDFR*, *MdANS*, and *MdUFGT* in apple peels and *VcCHS*, *VcCHI*, *VcF3H*, *VcDFR11*, *VcANS*, and *VcUFGT* in blueberry peels were analyzed using qRT-PCR [54]. Three biological replications were used for the anthocyanin content determination and qRT-PCR analysis.

### 4.9. DNA Isolation, VcDFR11 Promoter Cloning, and Yeast One Hybrid (Y1H)

By using a plant genomic DNA kit (Cat# GDP304) (Tiangen, Beijing, China), genomic DNA (gDNA) was isolated using ‘FL03’ blueberry leaves as materials. By using ‘FL03’ blueberry gDNA as template, a 206 bp (only containing bHLH binding site) and an approximately 2000 bp (containing binding sites for both bHLH and MYB TFs) long *VcDFR11* promoter sequences were individually amplified, gel purified, and subjected to sequencing verifications. Then, the gel-purified short and long *VcDFR11* promoter sequences, pVcDFR11_206_ and pVcDFR11_2000_, were individually ligated into the pAbAi vector to obtain recombinant pVcDFR11_206_ and pVcDFR11_2000_ vectors and transformed into yeast strain Y1HGold. For Y1H assay, pGADT7-VcAN1/VcbHLH42-1/VcbHLH1-1/VcbHLH1-2/VcMYB-1 (provided by our lab, Zhang et al. [25,51]) vectors were separately transformed into yeast strains Y1HGold-pVcDFR11_206_/VcDFR11_2000_ according to Zhang et al. [51].

### 4.10. Dual-Luciferase Assay (LUC)

By using a ready-to-use seamless cloning kit (Cat# B632219) (Sangon Biotech, Shanghai, China), the 2000 bp *VcDFR11* promoter sequences were introduced into the pNC-Green-LUC vector to obtain the *LUC-pVcDFR11_2000_* recombinant vector. CDSs of *VcMYB-1* and *VcbHLHs* were introduced into pNC-Green-SK to obtain the SK-VcMYB-1 and SK-VcAN1/bHLH42-1/bHLH1-1/bHLH1-2 recombinant vectors. These recombinant vectors were individually transformed into *A*. *tumefaciens* GV3101 (pSoup-p19) cells and cultured till the OD_600_ reached 1. Then, agrobacteria carrying SK-VcMYB-1/VcAN1/bHLH42-1/bHLH1-1/bHLH1-2 were individually equally volume mixed with agrobacteria carrying *LUC-VcDFR11_2000_*, kept still at room temperature for 20 min, and inoculated into fully expanded healthy mature leaves of six-week-old *N. benthamiana* plants. pNC-Green-LUC + pNC-Green-SK, SK-VcMYB-1/VcAN1/bHLH42-1/bHLH1-1/bHLH1-2 + pNC-Green-LUC, and pNC-Green-SK + *LUC-VcDFR11_2000_* inoculation combinations were also made. After inoculation, tobacco plants were kept in the dark at 25 °C for 2 d, removed to normal light conditions for another 2 d, and sprayed with 1 mM luciferin substrate solution and kept in the dark for 5 min. Then, tobacco leaves were harvested and observed under a Tanon 5200 system (Tanon Science & Technology Co., Ltd., Shanghai, China).

### 4.11. Firefly Luciferase Complementation Imaging (LCI)

In one of our previous studies [25], LCI was successfully applied to verify the interactions between VcMYB-1 and VcAN1. In this study, *VcbHLH42-1*/*bHLH1-1*/*bHLH1-2* was individually introduced into the pCAMBIA1300-cLUC vector to obtain the cLUC-VcbHLH42-1/bHLH1-1/bHLH1-2 recombinant vectors, transformed into *agrobacteria* GV3101 (pSoup-p19), and subjected to LCI assay to investigate the interactions between VcbHLH42-1/bHLH1-1/bHLH1-2 and VcMYB-1 according to Yan et al. [55].

### 4.12. Statistical Analyses

The anthocyanin contents and relative gene expression levels in different samples were presented as mean ± standard deviations of at least three replications. SPSS 22.0 (IBM corporation, Armonk, NY, USA) was used to analyze the difference significance of measured parameters among different samples at the *p* < 0.05 level. For figure drawing, GraphPad Prism 8 was used.

## 5. Conclusions

In this study, we functionally characterized two members (*VcDFR11* and *VcDFR30*) of the 36 blueberry *VcDFR* genes. The two of *VcDRFs*’ overexpression both significantly promote anthocyanin accumulation and upregulate corresponding structural genes’ expression (especially the downstream *ANS* and *UFGT* genes), with the former one showing much better anthocyanin-biosynthesis-promoting effects. Moreover, we found that the ‘MYB-bHLH-DFR’ module contributed greatly to anthocyanin biosynthesis in blueberries. Our study demonstrated the role of *VcDFR11* in anthocyanin biosynthesis and can provide basis for its application in blueberry genetic breeding.

## Data Availability

The data supporting reported results can be found in the manuscript and Supplemental Materials.

## Supplementary Materials

The following supporting information can be downloaded at: https://www.mdpi.com/article/10.3390/plants14101449/s1, Figure S1: similarity analysis results of blueberry *DFR* genes (A) and their encoded proteins (B). The redder the color, the higher the similarity, and the greener the color, the lower the similarity; Figure S2: LCI assay results for the interactions between VcMYB-1 and VcAN1 (A), VcbHLH42-1 (B), VcbHLH1-1 (C), and VcbHLH1-2 (D), respectively; Table S1: physiochemical properties of VcDFRs. AA: amino acid; pI: isoelectric point; GRAVY: grand average of hydropathicity; Table S2: information for the primers used in this study; Table S3: gene duplication analysis results of blueberry *DFR* genes. Mya: million years ago. For abbreviation, ‘VaccDscaff’ is removed from all gene IDs in this table.

## Abbreviations

The following abbreviations are used in this manuscript:

AA	Amino acid
ABA	Abscisic acid
ANS	Anthocyanindin synthase
BF	Blue fruit
bp	Base pair
CDS	Coding sequence
DFR	*Dihydroflavonol 4-reductase*
ET	Ethylene
GA	Gibberellin
GF	Green fruit
LCI	Firefly luciferase complementation imaging
LUC	Dual-luciferase assay
MeJA	Methyl jasmonate
PF	Purple fruit
PiF	Pink fruit
qRT-PCR	Quantitative real-time PCR
RF	Red fruit
RT-PCR	Reverse transcription PCR
SA	Salicylic acid
TF	Transcription factor
UFGT	UDP-glucose: flavonoid-3-*O*-glucosyltransferase
UTR	Untranslated region
Y1H	Yeast one hybrid
