# Supplementary material for: Functional Characterization of Anthocyanin Biosynthesis-Related Dihydroflavonol 4-reductase (DFR) Genes in Blueberries (Vaccinium corymbosum)
Source: Plants (Basel). 2025 May 13;14(10):1449. doi: 10.3390/plants14101449 (PMC12114909; doi:10.3390/plants14101449)
Supplement: Supplementary file 1 [file plants-14-01449-s001.zip › Supplemental Tables.pdf]

Supplemental Table S1 Physiochemical properties of VcDFRs. AA: amino acid; pI: isoelectric point; GRAVY: grand average of hydropathicity.

Gene name	Gene ID	Number of AA	Molecular Weight/Da	pI	Instability Index	Aliphatic Index	GRAVY	Subcellular localization
VcDFR1	1-snap-gene-300.40	340	37769.52	8.49	38.09	87.21	-0.279	Golg
VcDFR2	2-augustus-gene-301.26	330	36623.1	6.01	37.2	93.61	-0.081	Golg
VcDFR3	3-augustus-gene-111.27	303	33729.62	5.93	31.33	89.41	-0.163	Cyto
VcDFR4	4-processed-gene-398.7	355	39060.47	5.55	29.67	85.35	-0.254	Golg
VcDFR5	4-augustus-gene-398.17	329	36034.48	8.3	26.08	91.82	-0.138	Golg
VcDFR6	8-snap-gene-149.28	320	35226.52	7.06	38.43	87.78	-0.206	Chlo,Golg
VcDFR7	9-processed-gene-377.29	363	40076.68	5.9	30.57	85.34	-0.281	Golg
VcDFR8	9-snap-gene-378.39	518	56327.94	7.1	24.75	95.19	-0.034	Golg
VcDFR9	10-snap-gene-130.47	340	37606.3	7.59	37.4	88.06	-0.244	Cyto
VcDFR10	11-snap-gene-49.32	324	35681.84	6.72	34.3	90.22	-0.116	Golg
VcDFR11	13-processed-gene-166.8	345	38448.08	5.69	25.33	83.62	-0.187	Chlo,Golg
VcDFR12	13-snap-gene-317.64	322	36066.29	7.09	37.51	89.01	-0.199	Chlo,Golg
VcDFR13	14-augustus-gene-255.37	330	36623.1	6.01	37.2	93.61	-0.081	Golg
VcDFR14	15-snap-gene-57.22	324	35750.95	6.46	35.75	92.62	-0.085	Golg
VcDFR15	15-augustus-gene-178.21	333	36113.33	5.75	33.92	88.47	-0.008	Golg
VcDFR16	17-processed-gene-327.1	334	36580.92	5.94	36.09	88.98	-0.173	Golg
VcDFR17	19-augustus-gene-130.25	312	34406.43	5.7	37.32	92.44	-0.049	Golg
VcDFR18	19-snap-gene-152.16	324	35750.95	6.46	35.75	92.62	-0.085	Golg
VcDFR19	*19-augustus-gene-256.19*	*333*	*36134.39*	*5.84*	*32.79*	*89.64*	*0.009*	*Golg*
VcDFR20	20-snap-gene-127.45	328	36428.67	5.71	39.24	91.49	-0.084	Golg
VcDFR21	24-augustus-gene-201.24	355	38364.82	5.36	35.87	87.94	0.014	Chlo,Golg
VcDFR22	24-augustus-gene-324.25	324	36037.36	5.95	31.57	90.83	-0.159	Chlo,Golg
VcDFR23	24-snap-gene-325.25	324	35697.9	6.72	34.51	89.94	-0.117	Golg

VcDFR24	25-augustus-gene-296.41	330	36623.1	6.01	37.2	93.61	-0.081	Golg
VcDFR25	28-augustus-gene-219.28	282	31436.04	5.74	42.06	95.35	-0.011	Golg
VcDFR26	30-snap-gene-79.51	343	38601.35	7.09	37.37	88.95	-0.139	Chlo,Cyto
VcDFR27	32-snap-gene-13.33	343	38572.35	7.09	36.95	90.09	-0.116	Chlo
VcDFR28	32-augustus-gene-62.24	322	36066.29	7.09	37.51	89.01	-0.199	Chlo,Golg
VcDFR29	32-processed-gene-199.0	363	40560.6	5.7	25.94	86.47	-0.16	Golg
VcDFR30	35-processed-gene-293.6	363	40078.66	5.9	30.8	84.55	-0.295	Golg
VcDFR31	35-augustus-gene-293.13	329	36092.52	8.02	26.54	91.82	-0.147	Golg
VcDFR32	36-augustus-gene-33.15	309	33789.98	8.59	25.7	95.24	-0.089	Golg
VcDFR33	36-processed-gene-33.3	373	40868.98	6.76	28.32	92.47	-0.092	Golg
VcDFR34	42-processed-gene-96.9	363	40560.6	5.7	25.94	86.47	-0.16	Golg
VcDFR35	133-augustus-gene-0.14	333	36026.25	5.74	33.08	89.64	0.032	Golg
VcDFR36	1613-processed-gene-0.0	336	37801.6	6.09	33.27	85.57	-0.129	Golg

Supplemental Table S2 Information for the primers used in this study.

Target Name	Forward Primer	Reverse Primer	Applications
VcDFR11	ATGAAAGGTGTTAACAGTGGGCT	CTAAATCGTTCCATTTCCATTTCCATTG	Gene cloning
VcDFR30	ATGCCATCAGTTTCTGGCCAAAC	TCAAGAGTGAATGCGAATAATAG	
VcDFR11$_{206}$	GTGTGGTCGAAGTAGAAAG	AGCTAATAATTAAGGAGTGCAG	Promoter cloning
VcDFR11$_{2000}$	CGATGGTGGCACGACCTATAC	GACTGGTTCGAATATCAAATAAAACCCG	
VcDFR11-OE	ACGGGGGACTCTAGAGGATCCATGAAAGGTGTTAACAGTGGGCT	GCTCACCATCGCTGCACTAGTCTAAATCGTTCCATTTCCATTTCCATTG	Vector construction
VcDFR30-OE	ACGGGGGACTCTAGAGGATCCATGCCATCAGTTTCTGGCCAAAC	GCTCACCATCGCTGCACTAGTTCAAGAGTGAATGCGAATAATAG	
VcDFR11$_{206}$-Ai	AATTCGAGCTCGGTACCCGGGGTGTGGTCGAAGTAGAAAG	ATACAGAGCACATGCCTCGAGAGCTAATAATTAAGGAGTGCAG	
VcDFR11$_{2000}$-Ai	AATTCGAGCTCGGTACCCGGGCGATGGTGGCACGACCTATAC	ATACAGAGCACATGCCTCGAGGACTGGTTCGAATATCAAATAAAACCCG	
VcMYB-1-AD	GCCATGGAGGCCAGTGAATTCATGGACATAGTTCCATTGGGAG	CAGCTCGAGCTCGATGGATCCAATATCCCAAAGGTCCACATTGTC	
GAPDH	GCTCCCAGCAAGGATGCCCC	CGGAAGGCCATTCCAGTCAACT	qRT-PCR
qVcDFR11	GCTTCTTGAACGGGGCTATG	CTTCAATGGCCTCGTCGAAG	
qVcDFR30	GGAGCGGAGGAGAGACTAAC	TTTGTTCCAATCACTGCCGG	
VcCHS			
VcCHI			
VcF3H			
VcANS			
VcUFGT			
MdActin	TGACCGAATGAGCAAGGAAATTACT	TACTCAGCTTTGGCAATCCACATC	
qMdCHS	GGAGACAACTGGAGAAGGACTGGAA	CGACATTGATACTGGTGTCTTCA	
qMdCHI	GGGATAACCTCGCGGCCAAA	GCATCCATGCCGGAAGCTACAA	
qMdF3H	TGGAAGCTTGTGAGGACTGGGGT	CTCCTCCGATGGCAAATCAAAGA	
qMdDFR	GATAGGGTTTGAGTTCAAGTA	TCTCCTCAGCAGCCTCAGTTTTCT	
qMdANS	CCAAGTGAAGCGGGTTGTGCT	CAAAGCAGGCGGACAGGAGTAGC	

qMdUFGT	CCACCGCCCTTCCAAACACTCT	CACCCTTATGTTACGCGGCATGT

Supplemental Table S3 Gene duplication analysis results of blueberry DFR genes. Mya: million years ago. For abbreviation, 'VaccDscaff' is removed from all gene IDs in this table.

Gene ID	Gene Name	Gene ID	Gene Name	Ka	Ks	Ka_Ks	Duplication date/Mya	Duplication type
1-snap-gene-300.40	*VcDFR1*	8-snap-gene-149.28	*VcDFR6*	0.0096	0.0603	0.1587	0.23	Segmental duplication
1-snap-gene-300.40	*VcDFR1*	10-snap-gene-130.47	*VcDFR9*	0.0155	0.0392	0.3942	0.15	Segmental duplication
2-augustus-gene-301.26	*VcDFR2*	20-snap-gene-127.45	*VcDFR20*	0.0928	0.755	0.1229	2.9	Segmental duplication
2-augustus-gene-301.26	*VcDFR2*	25-augustus-gene-296.41	*VcDFR24*	0	0	-	0	Segmental duplication
2-augustus-gene-301.26	*VcDFR2*	3-augustus-gene-111.27	*VcDFR3*	0.0145	0.1164	0.1247	0.45	Segmental duplication
4-processed-gene-398.7	*VcDFR4*	9-processed-gene-377.29	*VcDFR7*	0.0049	0.0281	0.1758	0.11	Segmental duplication
4-augustus-gene-398.17	*VcDFR5*	9-snap-gene-378.39	*VcDFR8*	0.0179	0.0366	0.4881	0.14	Segmental duplication
10-snap-gene-130.47	*VcDFR9*	8-snap-gene-149.28	*VcDFR6*	0.0109	0.0556	0.197	0.21	Segmental duplication
11-snap-gene-49.32	*VcDFR10*	15-snap-gene-57.22	*VcDFR14*	0.0068	0.0132	0.5115	0.05	Segmental duplication
13-processed-gene-166.8	*VcDFR11*	32-processed-gene-199.0	*VcDFR29*	0.0025	0.0129	0.1936	0.05	Segmental duplication
13-processed-gene-166.8	*VcDFR11*	42-processed-gene-96.9	*VcDFR34*	0.0025	0.0173	0.1446	0.07	Segmental duplication
13-snap-gene-317.64	*VcDFR12*	30-snap-gene-79.51	*VcDFR26*	0.0075	0.0753	0.0998	0.29	Segmental duplication
13-snap-gene-317.64	*VcDFR12*	32-augustus-gene-62.24	*VcDFR28*	0	0	-	0	Segmental duplication
13-snap-gene-317.64	*VcDFR12*	32-snap-gene-13.33	*VcDFR27*	0.0068	0.0728	0.0939	0.28	Segmental duplication
14-augustus-gene-255.37	*VcDFR13*	19-augustus-gene-130.25	*VcDFR17*	0.08	0.6974	0.1147	2.68	Segmental duplication
14-augustus-gene-255.37	*VcDFR13*	2-augustus-gene-301.26	*VcDFR2*	0	0	-	0	Segmental duplication
14-augustus-gene-255.37	*VcDFR13*	20-snap-gene-127.45	*VcDFR20*	0.0928	0.755	0.1229	2.9	Segmental duplication
14-augustus-gene-255.37	*VcDFR13*	25-augustus-gene-296.41	*VcDFR24*	0	0	-	0	Segmental duplication

14-augustus-gene-255.37	*VcDFR13*	28-augustus-gene-219.28	*VcDFR25*	0.0881	0.7248	0.1215	2.79	Segmental duplication
14-augustus-gene-255.37	*VcDFR13*	3-augustus-gene-111.27	*VcDFR3*	0.0145	0.1164	0.1247	0.45	Segmental duplication
15-snap-gene-57.22	*VcDFR14*	24-snap-gene-325.25	*VcDFR23*	0.0095	0.0133	0.7146	0.05	Segmental duplication
15-augustus-gene-178.21	*VcDFR15*	19-augustus-gene-256.19	*VcDFR19*	0.0052	0.0311	0.1677	0.12	Segmental duplication
15-augustus-gene-178.21	*VcDFR15*	24-augustus-gene-201.24	*VcDFR21*	0	0.0356	0	0.14	Segmental duplication
17-processed-gene-327.1	*VcDFR16*	9-processed-gene-377.29	*VcDFR7*	0.0829	0.8586	0.0965	3.3	Segmental duplication
19-augustus-gene-130.25	*VcDFR17*	20-snap-gene-127.45	*VcDFR20*	0.0056	0.0182	0.3097	0.07	Segmental duplication
19-augustus-gene-130.25	*VcDFR17*	25-augustus-gene-296.41	*VcDFR24*	0.08	0.6974	0.1147	2.68	Segmental duplication
19-augustus-gene-130.25	*VcDFR17*	28-augustus-gene-219.28	*VcDFR25*	0.0033	0.0102	0.3276	0.04	Segmental duplication
19-augustus-gene-130.25	*VcDFR17*	3-augustus-gene-111.27	*VcDFR3*	0.0961	0.8004	0.12	3.08	Segmental duplication
19-snap-gene-152.16	*VcDFR18*	24-snap-gene-325.25	*VcDFR23*	0.0095	0.0133	0.7146	0.05	Segmental duplication
19-augustus-gene-256.19	*VcDFR19*	24-augustus-gene-201.24	*VcDFR21*	0.0052	0.0311	0.1677	0.12	Segmental duplication
20-snap-gene-127.45	*VcDFR20*	25-augustus-gene-296.41	*VcDFR24*	0.0928	0.755	0.1229	2.9	Segmental duplication
20-snap-gene-127.45	*VcDFR20*	28-augustus-gene-219.28	*VcDFR25*	0.0031	0.0245	0.1282	0.09	Segmental duplication
25-augustus-gene-296.41	*VcDFR24*	28-augustus-gene-219.28	*VcDFR25*	0.0881	0.7248	0.1215	2.79	Segmental duplication
25-augustus-gene-296.41	*VcDFR24*	3-augustus-gene-111.27	*VcDFR3*	0.0145	0.1164	0.1247	0.45	Segmental duplication
30-snap-gene-79.51	*VcDFR26*	32-augustus-gene-62.24	*VcDFR28*	0.0075	0.0753	0.0998	0.29	Segmental duplication
30-snap-gene-79.51	*VcDFR26*	32-snap-gene-13.33	*VcDFR27*	0.0051	0.042	0.1218	0.16	Segmental duplication
32-snap-gene-13.33	*VcDFR27*	32-augustus-gene-62.24	*VcDFR28*	0.0068	0.0728	0.0939	0.28	Segmental duplication
32-processed-gene-199.0	*VcDFR29*	42-processed-gene-96.9	*VcDFR34*	0	0.0122	0	0.05	Segmental duplication
35-processed-gene-293.6	*VcDFR30*	35-augustus-gene-293.13	*VcDFR31*	0.0952	0.3144	0.3028	1.21	Tandem duplication
35-processed-gene-293.6	*VcDFR30*	36-augustus-gene-33.15	*VcDFR32*	0.0889	0.298	0.2983	1.15	Segmental duplication
35-processed-gene-293.6	*VcDFR30*	4-processed-gene-398.7	*VcDFR4*	0.0025	0.0281	0.0877	0.11	Segmental duplication
35-processed-gene-293.6	*VcDFR30*	9-processed-gene-377.29	*VcDFR7*	0.0024	0.0397	0.0607	0.15	Segmental duplication
35-augustus-gene-293.13	*VcDFR31*	4-augustus-gene-398.17	*Vc DFR5*	0.0013	0	-	0	Segmental duplication
35-augustus-gene-293.13	*VcDFR31*	9-snap-gene-378.39	*VcDFR8*	0.0194	0.0367	0.5279	0.14	Segmental duplication

36-augustus-gene-33.15	*VcDFR32*	4-augustus-gene-398.17	*Vc DFR5*	0.0094	0.0332	0.283	0.13	Segmental duplication
36-augustus-gene-33.15	*VcDFR32*	9-snap-gene-378.39	*VcDFR8*	0.0241	0.0485	0.4979	0.19	Segmental duplication
36-processed-gene-33.3	*VcDFR33*	4-processed-gene-398.7	*VcDFR4*	0.0026	0.0208	0.1267	0.08	Segmental duplication
36-processed-gene-33.3	*VcDFR33*	9-processed-gene-377.29	*VcDFR7*	0.0039	0.0287	0.1346	0.11	Segmental duplication
133-augustus-gene-0.14	*VcDFR35*	15-augustus-gene-178.21	*VcDFR15*	0.0059	0.0516	0.114	0.2	Segmental duplication
133-augustus-gene-0.14	*VcDFR35*	19-augustus-gene-256.19	*VcDFR19*	0.0078	0.0493	0.159	0.19	Segmental duplication
133-augustus-gene-0.14	*VcDFR35*	24-augustus-gene-201.24	*VcDFR21*	0.0059	0.047	0.1252	0.18	Segmental duplication
