# Supplementary figures and images for: Functional Characterization of Anthocyanin Biosynthesis-Related Dihydroflavonol 4-reductase (DFR) Genes in Blueberries (Vaccinium corymbosum)
Source: Plants (Basel). 2025 May 13;14(10):1449. doi: 10.3390/plants14101449 (PMC12114909; doi:10.3390/plants14101449)

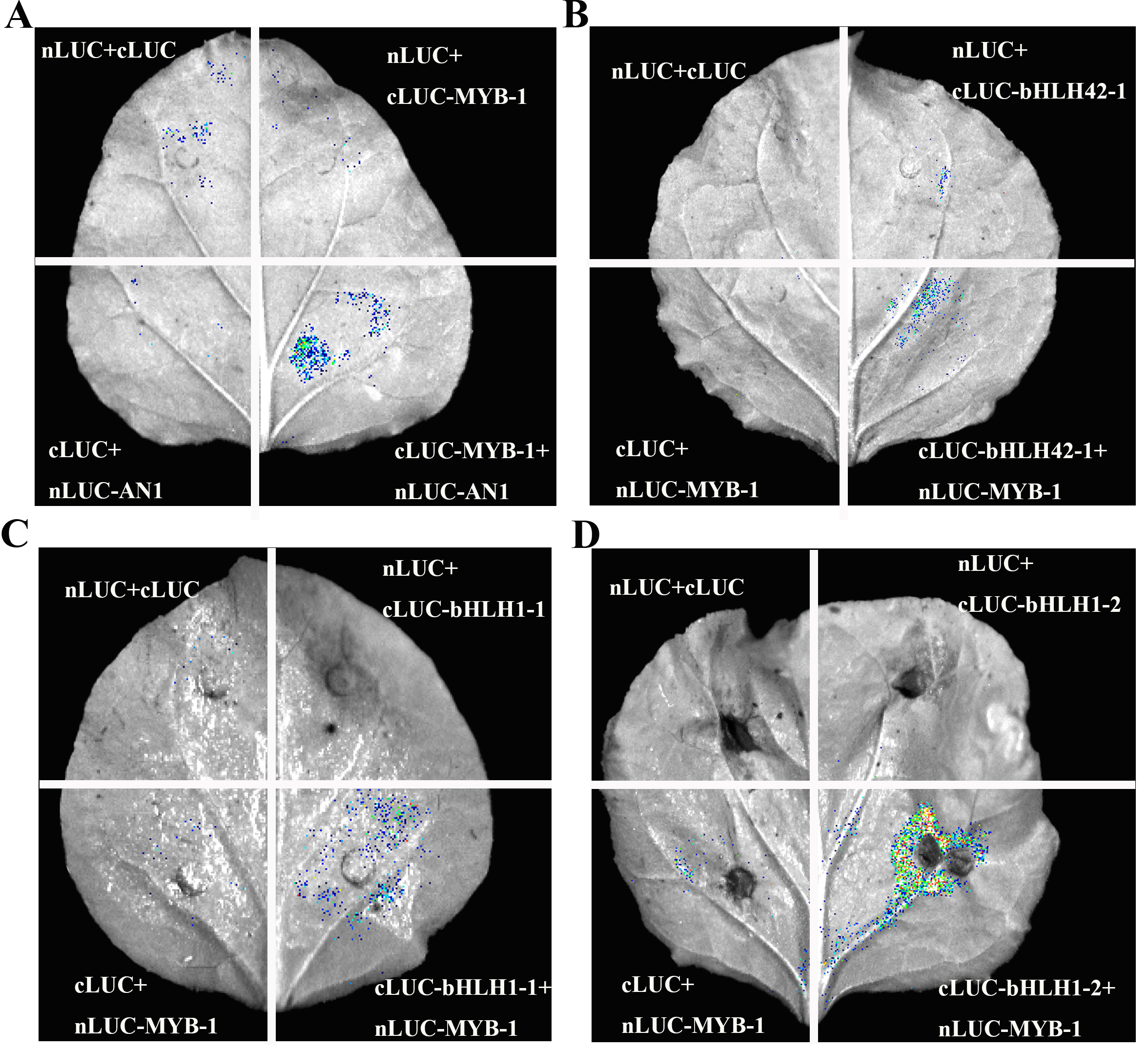

Supplement: Supplementary file 1 [file plants-14-01449-s001.zip › Figure S2.png]
